# 

**Published:** 1913-11

**Authors:** 


*OFFICIAL ORGAN—State Society Social Hygiene and Eugenics.*

VOL. XXIX　　NOVEMBER, 1913　　No. 5

### *The* "Red Ba[illegible]"

Surgeon General, U. S. A.,
Washington, D. C.

### TEXAS MEDICAL JOUR[illegible]AL

ESTABLISHED JULY, 1885

PUBLISHED MONTHLY AT AUSTIN, TEXAS, BY

F. E. DANIEL, M. D., - - - Editor and Proprietor.

PRINTED BY THE VON BOECKMANN-JONES CO.

**Subscription, $1.00 a Year, in Advance. - - - - Single Copies, 10 Cents**

Entered at the Postoffice at Austin, Texas, as second class mail matter.

